# Bond strength of a new Kevlar fiber-reinforced composite post with semi-interpenetrating polymer network (IPN) matrix

**DOI:** 10.4317/jced.55703

**Published:** 2019-08-01

**Authors:** Ahmed G. Almaroof, Samer A. Thyab, Ahmed H. Ali

**Affiliations:** 1 BDS, MSc and PhD (UK). Lecturer at Department of Aesthetic and Restorative Dentistry, College of Dentistry, University of Baghdad, Iraq; 2 BDS, MSc (Iraq). Lecturer at Department of Aesthetic and Restorative Dentistry, College of Dentistry, University of Baghdad, Iraq; 3 BDS, MSc and PhD (UK). Lecturer at Department of Aesthetic and Restorative Dentistry, College of Dentistry, University of Baghdad, Iraq

## Abstract

**Background:**

This study aimed to evaluate the bond strength and the penetration depth of two adhesive systems to a new experimental semi-IPN fiber post versus a commercial post.

**Material and Methods:**

Experimental Kevlar fiber (KF) and control everStick®POST (ES) posts (n=20/ group) with a diameter of 1.5 mm were used, 10 posts coated with StickResin (SR) and the other 10 posts coated with Scotch bond multipurpose (SBMP) adhesives. Composite resin buildup was performed over each post, using a cylindrical plastic mold (10 mm × 6 mm). Four discs of 2 mm thickness were prepared from each post/composite buildup and underwent pushout bond strength test at a crosshead speed of 0.5 mm/min accompanied by failure mode analysis. A further three specimens from each subgroup were bonded with adhesives labeled with 0.1 wt% Rhodamine B and embedded in acrylic resin, sectioned and examined under a confocal Laser-scanning microscope (CLSM) to measure the depth of resin penetration. Statistical analysis included ANOVA and Tukey test, the significance level was assumed at a p-value less than 0.05.

**Results:**

The push-out bond strength of KF was comparable to that of ES with both SBMP and SR adhesives (*P* >0.05). Bond strength value for SBMP was higher than SR adhesive in either ES and KF posts with no significant difference (*P* > 0.05). ES exhibited higher adhesive penetration depth compared with KF (*p*<0.05).

**Conclusions:**

The bond strength of Kevlar post was comparable with the everStick post and the semi-interpenetrating structure of Kevlar post displayed some adhesive monomers diffusion indicating its usefulness as a new intracanal post.

** Key words:**Kevlar fiber post, Bond strength, Penetration depth, Confocal, Semi-IPN polymer system.

## Introduction

With the increasing demands for aesthetic restoration, fiber-reinforced composite (FRC) posts have gained popularity over traditional metallic posts in the treatment of severely damaged root filled teeth. The adhesion between the fiber posts and the resin composite materials used for cementation and core build-up is crucial for the long-term success of adhesive post-core restorations (1). However, a reliable adhesion is the main concern with most of the currently used prefabricated FRC posts because they are designed to be passively retained into the root canal and have a smooth surface that restricts micromechanical interlocking with resin composites. Furthermore, their highly cross-linked polymer matrix is chemically inert with no interactive functional groups to bond resin cement and composite core material resulting in a poor adhesion (2). Therefore, different surface treatment techniques have been employed, facilitating both chemical and micromechanical retention, to improve the bond at the post-core and post-cement interfaces (3,4). However, the main disadvantages of these techniques are the risk of fiber damage at the surface with possible weakening effects on the stability and integrity of the posts as well as changes in post shape and fit within the canal. On the other hand, the applications of semi-IPN technology are promising and considered as an effective method to enhance the adhesion between the fiber post and resin composite materials through the ability of adhesive resin monomers to diffuse into the linear phase in semi-IPN structure (5,6). The bonding of resin adhesives to a composite core or fiber posts transfers loads from the restoration to the tooth. Without proper interlocking, the stresses debond the restoration and resulting in treatment failure as shown by previous in vitro studies (7,8).

In our previous study, a new post material based on the semi-interpenetrating polymer network (IPN) reinforced with Kevlar fibers was reported (9). The ability of Kevlar fibers to reinforce polymeric matrices prompted the study and were used to reinforce 2, 2-Bis [4- (2-hydroxy-3 methacryloyloxypropyl)-phenyl] propane (Bis-GMA) and tri-ethylene glycol dimethacrylate (TEGDMA)/ Polymethyl methacrylate (PMMA) semi-IPN matrix with titanium dioxide (TiO2) nanofiller composite and characterized to function as a new intracanal fiber post. The fabricated KF post materials showed superior flexural strength, higher fatigue limit, excellent radiopacity, and comparable water uptake and cytotoxicity to that of everStick®POST, a commercial IPN-glass fiber post material in current clinical use.

The aims of the present study were to evaluate the push-out bond strength and the penetration depth of two adhesive systems to the new experimental semi-IPN Kevlar fiber post versus a commercial post.

The first null hypothesis stated that there is no difference in push-out bond strength value between the experimental and control posts using two adhesives. The second null hypothesis stated that there are equal penetration depths of both adhesives in the experimental and control posts.

## Material and Methods

-Push-out bond strength 

The experimental KF posts were prepared from surface treated Kevlar fibers and Bis-GMA/TEGDMA/PMMA semi-IPN polymer matrix as described earlier by Almaroof *et al.* (9). A total of 40 posts with a diameter of 1.5 mm were used for the test; 20 ES (GC Corporation, Tokyo, Japan) and 20 KF posts both consisting of the semi-IPN matrix polymer. Ten posts from each post type were treated with Stick resin adhesive (GC-Stick Tech, Turku, Finland), whereas the other ten posts were treated with Adper Scotchbond™ multi-purpose plus (3M ESPE, St. Paul, MN, USA). The components of the posts and adhesive systems used in this study are summarized in  Table 1.

**Table 1:**
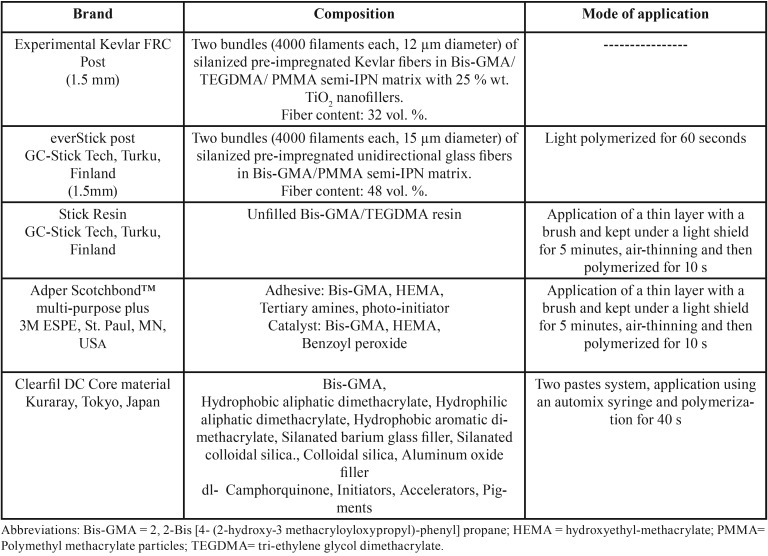
List of materials used in the study.

ES post groups were treated according to the manufacturers’ instructions. The posts were light polymerized for 60 seconds using a quartz-halogen-tungsten curing unit (Optilux 401, Kerr/Demetron, Orange, CA, USA), with a light intensity of 750 mW/cm². On both types of the tested posts, a layer of SR or SBMP adhesive was applied with a brush to cover 10 mm of the post length. Then posts were kept under a light shield for 5 minutes. The light protection was used to prevent polymerization of the light curable resin and to allow the monomers of the resin to diffuse into the polymer matrix. The layer of the bond was then thinned by gently blowing the surface of the posts with dry air and polymerized for 10s with the light curing unit. After light curing the adhesive resin, posts were centered vertically inside a cylindrical plastic mold (10 mm length, 6mm diameter) and Clearfil DC Core material (Kuraray, Tokyo, Japan) was injected to build up a block on the bonding surface. The composite then was light cured for 40s from each side and placed in relative humidity at 37◦C. After 24h, each block was transversely sectioned, using a slow speed, water cooled, diamond impregnated saw (Labcut 1010, Agar Scientific, Stansted, UK) to create four (2 mm thick) slices from each block for subsequent push-out bond strength tests (10), the thickness was verified using a digital electronic caliper, the top slices were discarded to avoid the influence of excess material. The following experimental groups were formed, Group 1: everStick post/StickResin (ES/SR), Group 2: Kevlar fiber post/StickResin (KF/SR), Group 3: everStick post/Scotchbond (ES/SBMP) and Group 4: Kevlar fiber post/Scotchbond (KF/SBMP).

**Figure 1:**
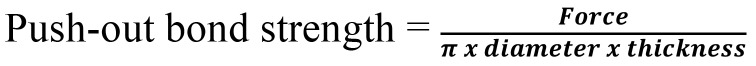
Formula.

Each slice was sufficiently supported by a stainless-steel jig with clearance for the dislodged post material. The push-out force was applied in an apical-coronal direction using a cylindrical plunger with a diameter of 1.4 mm attached to a universal testing machine (Instron model 5569A-Series Dual Column, High Wycombe, UK) at a crosshead speed of 0.5 mm/min until failure. The maximum load at failure was recorded in Newton (N) and was converted to MPa by dividing the applied load by the bonded area, using the following equation, (Fig. 1):

The modes of failure were examined visually using a stereomicroscope (WILD M32; Heerbrugg, Switzerland) at x30 and classified as adhesive failure between the post and the core material, cohesive failure within either the post or core material and mixed failure with partial interfacial adhesive failure with the presence of post/core cohesive failure.

-Adhesives penetration depth 

For this analysis, 0.1 wt.% Rhodamine B (Rh B: Sigma- Aldrich, UK) was added for both resin adhesives. A further three specimens from each group were bonded with the labeled adhesive systems, sectioned as described in the push-out bond strength section, and employed for the confocal microscopy analysis. (A total of eight sections were included in each experimental group).

The specimens were polished using wet silicon abrasive papers of ascending grit #600 to #2500 (Versocit; Struers Inc., Cleveland, OH, USA) with final ultra-sonication treatment in a distilled water bath for 5 min. The microscopy examination was performed using a confocal Laser-scanning microscope (Leica SP2; Leica, Heidelberg, Germany) equipped with a 63 x /1.4 NA oil-immersion lens and using 488-nm argon/helium (fluorescein excitation) or 568-nm krypton (rhodamine excitation) laser illumination.

**Figure 2:**
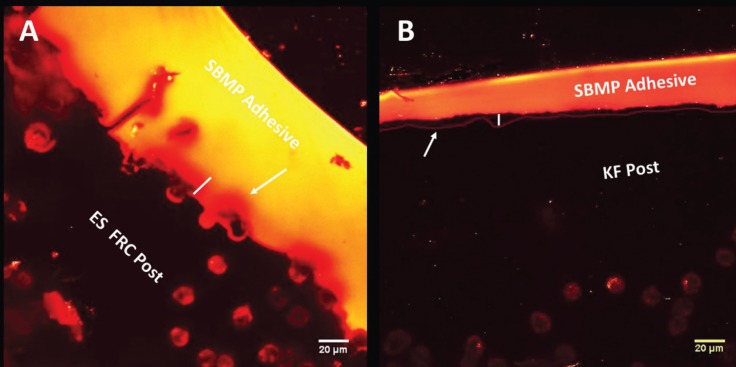
Confocal Laser scanning microscope image of A) ES (everStick®)/SBMP interface, B) KF (Kevlar Fiber)/SBMP interface. The white arrow indicates borderline of the inter-diffusion zone and the white line indicates the thickness of inter-diffusion zone.

The resin–post interface was investigated, and four images were recorded from each slice (32 images in total for each group). All images were further reconstructed with Image J software (ImageJ v1.41, U. S. National Institutes of Health, Bethesda, Maryland, USA). The examiner used a distinct borderline, which marked the outer boundary of the dissolved layer where the adhesive monomers dissolve the polymer and interlace into the interchain spacing, to measure the depths of interdiffusion from the images (Fig. 2). The dissolving depths along the diameter of the post were measured by one examiner at four sites in each image and the average was recorded.

-Statistical analysis 

After analyzing the normality of data distribution, a one-way ANOVA and Tukey’s post-hoc test were employed to detect the variances between the tested groups. The significance level was assumed at a *p*-value less than 0.05.

## Results

-Push out bond strength

The Mean ± standard deviation [SD] of the push-out bond values and the percentages of the failure modes shown in  Table 2. The mean push-out bond strength of the experimental KF post was statistically comparable to that of the commercial control ES post using SBMP or SR adhesive system (*p*>0.05). However, the mean push-out bond strength of the experimental KF post bonded with SR adhesive was statistically lower than that of the control ES post bonded with SBMP adhesive system (*p*<0.05), as shown in  Table 2.

**Table 2:**
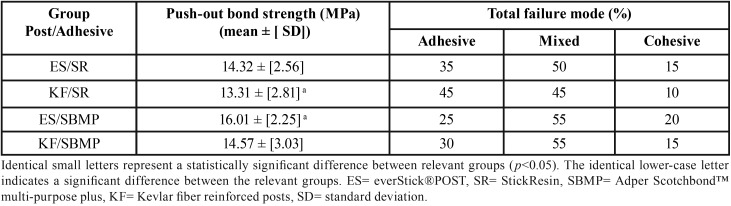
Push-out bond strength values (mean ± [SD]) and the percentages of failure modes (n=40).

The mean push-out bond strength values of SBMP adhesive was higher than that recorded for SR adhesive; however, within the same post group (i.e. either in KF groups or in ES groups), there were no significant differences between both adhesives. The failure mode analysis showed that most of the specimens failed mainly in mixed mode; however, some specimens exhibited post cohesive mode of failure. ES/SBMP bond showed the highest cohesive mode of failure (20%) compared to other groups. Also, KF/SR group showed the highest adhesive mode of failure (45%), as shown in  Table 2.

-Adhesive penetration depth

The morphological patterns of adhesive penetration are represented in Fig 2; the more continuous and homogenous dissolving layer of different thickness was seen in the ES posts groups. The results of adhesive penetration depth showed that the type of adhesive bond does not affect penetration depth, but the type of post affects the penetration depth significantly. There were no significant differences (*p*>0.05) in the penetration depth between ES/SR and ES/SBMP groups. Also, there was no significant difference (*p*>0.05) in the penetration depth between KF/SR and KF/SBMP groups. However, there were significant differences (*p*<0.05) in the penetration depth between ES/SBMP and KF/SR and KF/SBMP, respectively. Also, there were significant differences (*p*<0.05) in the penetration depth between ES/SR and KF/SR groups, as shown in  Table 3.

**Table 3:**
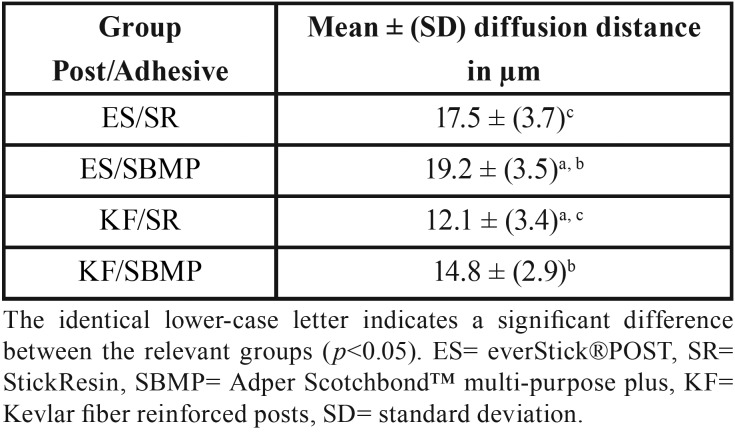
Mean thickness (mean ± [DS]) of inter-diffusion zone formed at posts/adhesives interface after 5 min immersion (n=12).

## Discussion

The matrix of KF post, a novel material based on the semi-IPN system, is formed by a combination of thermoplastic and thermoset polymers, which contains PMMA as a linear phase and Bis-GMA as a cross-linked phase. The matrix of the everStick post is based on the same system but in a pre-polymerizing plastic state and reinforced with silanated glass fibers. In literature, the bonding ability and the degree of bonding resins penetration of everStick post have been previously investigated. Studies have shown increased bond strength compared to other cross-linked FRC posts and there was some resin penetration into the surface of everStick posts (11-13), therefore, it was selected as a control in this study.

A layer of light curing resin adhesives (SR or SBMP) was applied and kept on the surface of both posts for 5 minutes before curing. The selection of this contact time was based on the previous study, which found that resin penetration was always noted after 300 s contact for semi-IPN posts (12). The push-out test was utilized to evaluate the bond strength of KF and ES posts to resin composite core using the two different adhesives. This test has been accepted as a reliable method for measuring the adhesion of endodontic posts as fracture occurs in parallel (not transverse) with the bonding interface, which simulates the clinical conditions (14). The aim of this investigation was to evaluate the bond strength between posts and the resin core materials in an optimum standardized way, while excluding the composite-dentine interface, therefore, the adhesively bonded posts were inserted into plastic tubes and surrounded by the composite core material.

The results from this study demonstrated that the push-out bond strength values obtained from KF posts were comparable to those of the commercial reference ES post when either SBMP or SR bond was used, leading to the acceptance of the first null hypothesis. The bond strength values were also within the range of previous studies on fiber post adhesion using different dental adhesives with resin composite materials (15,16), indicating the feasibility of applying the semi-IPN structure in FRCs as a new option to develop posts with improved bonding properties. With that in mind, the bond strength results observed with everStick posts being slightly higher than those observed with KF post may be related to the differences in surface structure and compositions of these two posts. Factors such as the structure of semi-IPN matrix (6), fiber type (17) and fiber content (18) have been found to have an influence on the bond strength of different FRC posts.

All the groups showed the mostly two types of failures including adhesive failures between the post and the core material and the cohesive failures within the core material and the post. Besides, complete adhesive or cohesive failures were also observed. From the results of the KF post, SBMP resulted in high bond strength than SR adhesive; similar results were also observed with ES post. However, the differences were not statistically significant. It has been reported that adhesive resin monomers with suitable dissolving parameters can diffuse into the linear phase, PMMA, of semi-IPN post matrix, improving the chemical adhesion between the surface of the post and resin composite materials (12,19). Bis-GMA monomer, which found within the composition of both SBMP and SR adhesives, has been shown to be capable of dissolving the linear phase in these materials (20). The presence of hydroxyethyl methacrylate (HEMA) within the composition of SBMP may enhance its diffusion capabilities, increasing the opportunity to establish a good bonding over SR groups. A similar finding was reported by Wolff *et al.* (19) and Lastumäki *et al.* (21). They showed that HEMA can effectively dissolve the linear phases of semi-IPN, thus enhancing bond strength.

Here, CLSM imaging was selected to measure the interdiffusion depths of adhesive monomers. This technique is more reliable than other microscopic methods, which enabled tracing the monomer diffusion into the post matrix polymer using a fluorescent dye (12). The observation obtained from CLMS images confirmed that the adhesive monomers were able to diffuse into the semi-IPN matrix of both tested posts (Fig. 1), however, the adhesive type had no significant effect on the penetration depth. SBMP diffused deeper than SR adhesive but did not differ significantly.

This study also showed that the differences in the penetration depth between ES and KF posts were highly significant for both adhesives, therefore the second hypothesis was rejected. A continuous and homogenous dissolving layer observed for ES post images, and both adhesives displayed a greater diffusion depth into the matrix of ES post when compared to KF post. However, it seems that the thickness of inter-diffusion zone formed at posts/adhesives interface had no significant effects on bond strength value. Although these highly significant differences in penetration depth between the two posts, their bond strength values were statistically comparable. This finding suggests that, irrespective to its depth, the occurrence of some resin diffusion into the surface of semi-IPN posts lead to an adequate adhesion between the bonded materials.

ES post bonded with SBMP exhibited the highest bond strength and more cohesive failures among other groups, while KF post bonded with SR exhibited the lowest bond strength and failed mostly in adhesive mode. It has been reported that the in-situ light-polymerization of the everStick® post, which resulted in a lower degree of conversion and cross-linking density, would provide a better bonding facility by enhancing the diffusion of adhesive monomer into the resinous matrix of the post (18).

There was almost no difference in push-out force between Kevlar and everStick posts, the semi-IPN polymer-based posts. Kevlar fiber posts exhibited an appropriate bonding ability to resin composite core material indicating its successfulness as new intracanal post materials. The ability of dental adhesives to penetrate the surface of semi-IPN matrix-based posts is influenced by the structure and the constituents of this matrix. Proper selection of adhesive resin may enhance the interfacial adhesion between these posts and the resin composite core materials, which can apparently influence the clinical longevity of a post-core system.
